# Delirium Tremens

**Published:** 1843-05-27

**Authors:** 


### DELIRIUM TREMENS.

M. Sharn has tried, with the greatest benefit, preparations of ammonia in the treatment of delirium tremens.—*Ibid.*

